# Effects of Dietary *Phaffia rhodozyma* Astaxanthin on Growth Performance, Carotenoid Analysis, Biochemical and Immune-Physiological Parameters, Intestinal Microbiota, and Disease Resistance in *Penaeus monodon*

**DOI:** 10.3389/fmicb.2021.762689

**Published:** 2021-11-03

**Authors:** Weilong Wang, Mengting Liu, Samia Fawzy, Yucai Xue, Meiqin Wu, Xuxiong Huang, Ganfeng Yi, Qian Lin

**Affiliations:** ^1^Centre for Research on Environmental Ecology and Fish Nutrition of the Ministry of Agriculture, Shanghai Ocean University, Shanghai, China; ^2^Institute of Bast Fiber Crops, Chinese Academy of Agricultural Sciences, Changsha, China; ^3^Shanghai Collaborative Innovation for Aquatic Animal Genetics and Breeding, Shanghai Ocean University, Shanghai, China; ^4^National Demonstration Center for Experimental Fisheries Science Education, Shanghai Ocean University, Shanghai, China; ^5^College of Marine Ecology and Environment, Shanghai Ocean University, Shanghai, China; ^6^Beijing Dabeinong Technology Group Co., Ltd., Beijing, China

**Keywords:** astaxanthin, *Phaffia rhodozyma*, *Penaeus monodon*, shrimp health, intestinal microbiota

## Abstract

The present study aimed to investigate the effect of dietary astaxanthin (Ast) from *Phaffia rhodozyma* on growth performance, survival, carotenoid content, the activity of antioxidant and immune-related enzymes, intestinal microbiota comparison, and disease resistance against *Vibrio parahaemolyticus* in *Penaeus monodon*. Juveniles (average weight 3.15 ± 0.12 g) were fed with six experimental diets supplemented with 0 (Control), 20.5, 41, 61.5, 82, and 102.5 mg/kg of Ast (defined as diet A–D) in triplicate for 56 days. The results indicated that shrimp fed with Ast supplementation significantly (*p* < 0.05) improved growth performance compared with the control. Furthermore, significantly (*p* < 0.05) increased survival and decreased feed conversion ratio (FCR) demonstrated the beneficial effects of dietary Ast on enhancing nutrient utilization and ultimately improving the growth and survival of shrimp. Furthermore, shrimp fed with Ast including diet developed a deeper red color than the control, consistent with the significantly (*p* < 0.05) increased Ast deposition in the shrimp shell. Hemolymph-immunological parameters [aspartate aminotransferase (AST), alanine aminotransferase (ALT), and alkaline phosphatase (AKP)] and hepatopancreatic antioxidant status [total antioxidant capacity (T-AOC), malondialdehyde (MDA), catalase (CAT), and superoxide dismutase (SOD)] were significantly (*p* < 0.05) affected by dietary Ast supplementation. Dietary increasing Ast levels significantly (*p* < 0.05) increased shrimp resistance performance to *V. parahaemolyticus* according to the LT_50_ results in the current study, which may be caused by increased total carotenoid contents in shrimp tissues from all the Ast-supplemented treatments. Conversely, intestinal microbiota biodiversity and richness were not affected by dietary Ast. The best performances of growth, antioxidant status, immunological response, and carotenoid deposition were observed in diets E and F among all the Ast-supplemented treatments. Overall, all the data suggested that dietary *P. rhodozyma* Ast played a critical role in improving growth performance, achieving the desired coloration, increasing carotenoid content, and keeping better health status of shrimp. Based on these positive performances, *P. rhodozyma* Ast could gain the trust of the consumers as a natural source and provide a potential alternative for synthetic Ast using in the *Penaeus monodon* culture industry.

## Introduction

The black tiger shrimp *Penaeus monodon*, one of the primary farmed penaeid shrimp species worldwide, is widely distributed on the southeastern coast of China. Consumers greatly favor *P. monodon* due to its rich nutrition and delicious taste, increasing the demand gradually. In recent years, due to the shortage of water and land resources, intensive farming has developed rapidly to meet the needs of the market (Biao and Yu, 2007; Zhang et al., 2017). However, higher feeding densities and more significant feed inputs come with a series of problems, such as water deterioration, disease outbreaks, and lightening of body color (Chien and Jeng, 1992; Verdegem, 2013; Bossier and Ekasari, 2017; Dauda, 2020). During intensive cultivation, the fluctuating pond environments may produce various environmental stressors (e.g., hypoxia, high concentration of ammonia, and nitrite), suppressing the antioxidant defense system and rendering shrimp susceptible to different diseases (Zhang et al., 2013). Meanwhile, outbreaks of pathogenic bacteria and viral diseases would negatively affect the growth and survival, led to massive economic losses, and impede the development of *P. monodon* aquaculture (Soonthornchai et al., 2009; Han et al., 2015). The color of shrimp flesh is an important indicator that could evaluate the health status and quality, directly affecting the choices of customers in the supermarket (Brun and Vidal, 2006). Since crustaceans themselves cannot synthesize pigment, the type, amount, and duration of pigments in the feed determine the color of the flesh and surface (Wade et al., 2017). Therefore, it is crucial to supplement antioxidants and pigments to prevent and manage the possible industrial problems during intensive aquaculture.

Astaxanthin (Ast) is a kind of symmetric ketocarotenoid, referred to as xanthophyll carotenoid, the end product of carotenoid synthesis, and distributes widely throughout nature (Pashkow et al., 2008). Because of containing ketone functional groups and hydroxyl, this compound owns several biological properties, including antioxidant action, immunomodulatory effects, anti-inflammatory properties, disease prevention, and coloration effects (Pashkow et al., 2008; Takahashi et al., 2011). Early studies found that dietary Ast could increase survival, enhance the antioxidant capacity, and improve the growth performance of *P. monodon* (Torrissen, 1995; Chien et al., 2003). Several reports had demonstrated that dietary Ast could significantly enhance shrimp tolerance to low temperature, low salinity, hypoxia, and ammonia stress (Chien et al., 2003; Pan et al., 2003; Chien and Shiau, 2005; Flores et al., 2007; Niu et al., 2009). In addition, supplementation of Ast in feed can darken the body color of aquatic animals, such as *Oncorhynchus mykiss* (Mora et al., 2006), *Amphilophus citrinellus* × *Cichlasoma synspilum* (Li et al., 2018), *Portunus trituberculatus* (Han et al., 2018), and *Marsupenaeus japonicus* (Wang et al., 2018b).

Ast is synthesized *de novo* by microbes, microalgae, and some plants in nature. Commercial products are mainly derived from natural sources and chemical synthesis (Ho et al., 2018; Jin et al., 2018). *Phaffia rhodozyma*, a red yeast, is one of the important natural sources of Ast (Andrewes et al., 1976; Lorenz and Cysewski, 2000). Because of the advantages of having a culture cycle, primarily utilizing sucrose, glucose as carbon sources, fermentation in high-density conditions, and not turn into toxic vitamin A in the body, *P. rhodozyma* is a potential Ast source with a commercial value that would be widely used in aquaculture (Mata-Gomez et al., 2014). In recent years, Ast from *P. rhodozyma* has been utilized to promote growth performance, antioxidant activities, quality of the meat, and the anti-inflammatory response of animals (Takahashi et al., 2011; Perenlei et al., 2015), such as finishing pigs (Yang, 2006), laying hens (Lorenz and Cysewski, 2000; Akiba et al., 2008), rainbow trout (Nakano et al., 1999). Nevertheless, no studies have focused on the effects of dietary *P. rhodozyma* on *P. monodon*. Therefore, the purpose of the present work was to investigate the effect of Ast from *P. rhodozyma* on growth performance, non-specific immune response, intestinal microbiota, and disease resistance against *Vibrio parahaemolyticus* in *P. monodon*.

## Materials and Methods

### Preparation of Astaxanthin and Experimental Diet Formulation

*Phaffia rhodozyma* (Ast containing: 0.41% of dry matter, produced by Lida Biotechnology Co., Ltd, Weihai, China) was used as natural Ast source for the current study.

Six iso-nitrogenous and iso-lipidic experimental diets were formulated with graded levels (0, 20.5, 41, 61.5, 82, and 102.5 mg/kg diet) of Ast (Table 1, defined as A–D, respectively). Fish meal, soybean meal, and peanut meal were added to cover the protein requirements of juveniles. Fish oil provided the primary lipid. Squid paste and soybean lecithin oil were used as the source of cholesterol and phospholipids. In addition, other ingredients were added to meet the essential requirements for juvenile shrimp. The basal composition (9.51% moisture, 45.68% crude protein, 5.50% crude lipid, and 11.52% ash) and Ast contents (1.41%) of the experimental diets were described in Table 2. All the dry ingredients for the experimental diets were mixed thoroughly after being ground and passed through an 80-mesh sieve. Lipid substances were added while blending to form a homogeneous mixture. After that, an appropriate amount of water was added to form a homogeneous dough. Then, the dough was made into pellets by passing through a 1.5 mm orifice and oven-dried at 40°C until the moisture content was reduced to 10%. The dried experimental diets were stored in a –20°C freezer until use.

**TABLE 1:** Formulation and composition of the experimental diets (%, dry matter basis).

**Ingredients**	**Treatments (Ast contents, mg/kg diet)**					
	**Control (A)**	**Ast_20_._5_ (B)**	**Ast_41_ (C)**	**Ast_61_._5_ (D)**	**Ast_82_ (E)**	**Ast_102_._5_ (F)**
Fish meal^a^	29	29	29	29	29	29
Soybean meal^a^	25	25	25	25	25	25
Peanut meal^a^	18	18	18	18	18	18
α-Starch^a^	5	5	5	5	5	5
Squid paste^a^	3	3	3	3	3	3
Fish oil^a^	2	2	2	2	2	2
Soybean lecithin^a^	2	2	2	2	2	2
Vitamin mixture^b^	0.4	0.4	0.4	0.4	0.4	0.4
Mineral mixture^c^	0.5	0.5	0.5	0.5	0.5	0.5
Vitamin C phosphate^a^	0.1	0.1	0.1	0.1	0.1	0.1
Monocalcium phosphate^a^	1	1	1	1	1	1
Wheat flour^a^	14	13.5	13	12.5	12	11.5
*Phaffia rhodozyma* ^d^	0	0.5	1	1.5	2	2.5
Total	100	100	100	100	100	100

*^*a*^Dabeinong Technology Group Co., Ltd, Beijing, China.*

*^*b*^Vitamin mixture: vitamin A (IU/kg), 1,200,000–6,800,000; vitamin D_3_, 500,000–1,000,000; vitamin E, 25,000–130,000; vitamin K_3_ (g/kg), ≥10; nicotinamide, ≥18.5; inositol, 100; folic acid, 2; vitamin B_12_ (mg/kg), 21; biotin, ≥ 240.*

*^*c*^Mineral mixture: calcium pantothenate (g/kg),12.5; Cu, 5–16.6; Fe, 25–100; Zn, 15–50; Mn, 2–20; Mg, 30–100; I (mg/kg), 100–1,000; Se, 10–166; Co, 100–166.*

*^*d*^*Phaffia rhodozyma* powder: Bairihong^®^ containing 0.41% (measured value) astaxanthin made by Lida Biotechnology Co., Ltd, Weihai, China. Astaxanthin comprised 83% of the total carotenoids.*

**TABLE 2:** Proximate and carotenoid composition of the experimental diets.

**Parameters** ^a^	**Treatments**					
	**A**	**B**	**C**	**D**	**E**	**F**
**Basal composition (%)**						
Crude protein	45.68 ± 0.43	44.86 ± 0.27	45.11 ± 0.18	46.05 ± 0.06	45.58 ± 0.24	45.99 ± 0.16
Crude lipid	5.50 ± 0.16	4.95 ± 0.31	5.03 ± 0.12	5.19 ± 0.11	5.38 ± 0.16	5.24 ± 0.07
Ash	11.52 ± 0.88	11.15 ± 0.05	10.60 ± 0.53	11.78 ± 0.29	12.43 ± 0.22	10.23 ± 0.75
Moisture	9.51 ± 0.61	10.15 ± 0.06	9.89 ± 0.05	10.89 ± 0.14	11.34 ± 0.09	11.65 ± 0.21
Astaxanthin (mg/kg)	1.41 ± 0.14	21.82 ± 0.37	40.37 ± 0.59	63.73 ± 0.19	83.15 ± 1.15	105.32 ± 0.31

*^*a*^Data are expressed as mean ± SEM from triplicate groups. ND, not detected.*

### Shrimp and Experimental Conditions

*Penaeus monodon* was supplied by Dabeinong Technology Group Co., Ltd, Beijing, China. Before the experiments, juveniles were maintained at an ambient water temperature (29 ± 0.5°C) in a cement pool (Length = 10 m, Width = 5m, and Height = 1.5 m), and fed with a commercial diet (40% crude protein and 8% crude lipid) to acclimatize the laboratory environment for 2 weeks. At the start of the feeding trial, 900 juveniles of similar size (3.15 ± 0.12 g) were randomly distributed into 18 polyethylene circular tanks (*H* = 117 cm and *R* = 50 cm), connected with a recirculating aquaculture system. During the feeding trial, experimental tanks were continuously aerated. The rearing water was supplied directly from the sea with salinity 28–30‰ after filtered and UV sterilized before use. Each tank was covered with a net to prevent the shrimp from jumping out. Juveniles were fed five times a day (7:00, 11:00, 15:00, 19:00, and 23:00) at a daily ration of 5–8% of body weight for 8 weeks. Uneaten feed was collected 3 h after feeding to calculate feed intake and feed efficiency ratio, while fecal matter was siphoned from the tanks. The experimental water was changed about 20% every 3 days to keep the water quality, including temperature, ammonia nitrogen, pH, and dissolved oxygen, were measured every day and kept at 28–31°C, < 0.05 mg/L, 8.0–8.5, and > 5 mg/L, respectively.

### Sampling Techniques

After the 8-week culture experiment, all the shrimp were fasted for 1 day before final sampling, recorded, and weighed all surviving shrimp and body weight from each tank. Five juveniles from each tank were obtained for whole-body composition and carotenoid analysis. Hemolymph was drawn with 1 ml sterile syringes from the first abdominal segment of 10 juveniles per tank; hepatopancreas was collected separately in 5 ml tubes from the 10 shrimp and stored at –80°C together with hemolymph samples for enzymatic activity analysis. Three mid-guts were randomly sampled, loaded into dividual 1.2 ml RNase-free cryogenic vials, and immediately stored in liquid nitrogen for intestinal microflora analysis.

### Biochemical Analysis

Moisture, crude protein, ash, and total lipid contents of experimental diets and whole-body shrimp were quantified per the Association of Official Analytical Chemists (AOAC, 2006). To measure the moisture content, each sample was dried in an oven to a constant weight at 105°C. The crude protein of the samples was determined by the Kjeldahl method using an Auto Kjeldahl System (2300-Auto-analyzer, Foss Tecator, Sweden). Ash content of the samples was carried out by using a muffle furnace at 550°C for 8 h. The crude lipid was determined by the ether extraction method using Soxtec System HT (Soxtec System HT6, Sweden) (Wang et al., 2018b).

Hemolymph samples were centrifuged at 4,000 rpm, at 4°C for 10 min, and the serum was separated and collected from supernatants. Weighted hepatopancreas samples were homogenized with shrimp saline solution in an ice-water bath (Wang et al., 2019). After total protein (TP) content was quantified, serum and hepatopancreas samples were used to determine the activities of antioxidant (superoxide dismutase SOD, catalase CAT, total antioxidant capacity T-AOC, and malondialdehyde MDA), and non-specific immune enzymes (aspartate aminotransferase AST, alanine aminotransferase ALT, and alkaline phosphatase AKP). According to the instructions of the manufacturer, all parameters were measured using the corresponding reagent kits purchased from Nanjing Jiancheng Biological Company Research Institute.

### Carotenoid Analysis

The shrimp samples were prepared into the shell (including carapace, telson, and uropod), muscle, and whole body. The samples were freeze dried and ground into a powder. Dried powder of shell (1 g), muscle (2 g), and whole body (1.5 g) per each treatment were weighed and placed into a 50-ml polypropylene centrifuge tube in triplicate. The carotenoid extraction process was conducted with chloroform by shaking for 10 min, then centrifuged at 4°C at 10,000 rpm for 5 min. All extraction steps were repeated three times for each sample until no more color was extracted. The extracts were collected and combined into new tubes, then dried in a rotary vacuum evaporator (Eyela SB 1100, Japan). After that, the carotenoids were dissolved in 8 ml acetone solution. Three milliliters of the solution was used for determining the total carotenoid concentration with a spectrophotometer (Puxi T6, China) at 475 nm (Johnston et al., 2000). The leftover 5 ml was dried by N_2_ and redissolved in 2 ml of mobile phase solution (Acetonitrile: Methanol, 70:30 v/v) for Ast analysis. After being filtered through 0.2-μm hydrophilic polypropylene disks (Pall Corp), all samples were analyzed with an Ultra Performance Liquid Chromatography (UPLC, Waters ACQUITY, United States) system at the same time. Ten microliters of the sample was injected into a Waters ACQUITY H-Class BEH C18 column (1.7 μm, 2.1 mm × 150 mm). The sample was delivered to the column using a linear gradient mobile phase consisting of A (100% dH_2_0), B (acetonitrile: methanol, 70:30 v/v), and C (100% methyl tert-butyl ether) at a flow rate of 0.2 ml/min. Relative amounts of carotenoids were identified by an ultraviolet/visible detector set to 475 nm and quantified based on commercially available standards (Ast, zeaxanthin, canthaxanthin, β-cryptoxanthin, echinenone, and β-carotene) by calculating the area under the curve of each peak. The Ast, canthaxanthin, zeaxanthin, echinenone, and β-carotene standards were purchased from Sigma-Aldrich (United States), and the β-cryptoxanthin standard was purchased from CaroteNature (Swit).

### Microbial Diversity Analysis of Intestines in the Shrimp

DNA was extracted from all samples using the MagPure Soil DNA LQ Kit (Magen D6356-02, China) following the instructions of the manufacturer. To amplify the V3–V4 hypervariable regions of the bacterial 16S rRNA gene for Illumina deep sequencing, universal primer pairs (343F: 5′-TACGGRAGGCAGCAG-3′ and 798R: 5′-AGGGTATCTAATCCT-3′) were used. PCR (Bio-Rad 580BR10905, United States) was performed in triplicate 30-μl reactional mixtures (containing 15 μl 2 × Gflex PCR Buffer, 1 μl of each primer, 2 μl template DNA, 0.6 μl of Tks Gflex DNA Polymerase, and ddH_2_O) at 94°C for 5 min, followed by 26 cycles at 94°C for 30 s, 56°C for 30 s, 72°C for 20 s, and a final extension at 72°C for 5 min. After checking the amplicon quality using gel electrophoresis, the PCR products were purified with Agencourt AMPure XP beads (Beckman Coulter Co., United States) and quantified using Qubit dsDNA assay kit (Life Technologies Q32854, United States). Sequencing was performed after adjusting the concentration using an Illumina MiSeq platform (OE Biotech Company, China) with two paired-end read cycles of 250 bases. The raw reads were deposited into the NCBI Sequence Read Archive (SRA) database (Accession Number: PRJNA761235).

The bioinformatic analysis in the current study was completed using the Oe-biotech bio-cloud platform^1^. Raw FASTQ files were preprocessed using Trimmomatic software (Bolger et al., 2014) to detect and cut off ambiguous bases (N) by cutting off low-quality sequences with an average quality score below 20 using a sliding window trimming approach. After trimming, FLASH (version 1.2.7) merged with paired-end reads: 10 bp of minimal overlapping, 200 bp of maximum overlapping, and 20% of maximum mismatch rate. After that, sequences were performed further denoising with the following criteria: abandoning ambiguous, homologous sequences or below 200 bp; retaining reads above 75% of Q20 using QIIME software (version 1.8.0); detecting and removing the reads with chimera using UCHIME (Edgar et al., 2011). Subsequently, the sequences were subjected to primer sequences for removing and clustering to generate operational taxonomic units (OTUs) using VSEARCH software at 97% similarity (Rognes et al., 2016). Taxonomy was assigned to all OUTs by annotated and blasted against the Sliva database (version 132) using the RDP classifier (confidence threshold was 70%).

### Susceptibility to *Vibrio parahaemolyticus*

The *V. parahaemolyticus* strains were obtained from Beijing Dabeinong Technology Group Co., Ltd. The bacteria were activated by infecting the *P. monodon.* Hemolymph samples from diseased juveniles were inoculated on TCBS agar culture medium plates to obtain virulent clones for the formal infection test per Yan et al. (2020). After a 56-day feeding trial, 10 juveniles with similar size from each tank were selected and injected with 20 μl of activated *V. parahaemolyticus* (1.2 × 10^8^CFU/ml). Continuous aeration and constant water temperature (29–30°C) were kept during the stress test that lasted for 1 week. The time to death was recorded, and mortality values were expressed as the LT_50_ per the method of Wang et al. (2018b).

### Calculations and Statistical Analysis

The indexes were calculated using the following formulas:

Survival (%) = (final number of shrimp/initial number of shrimp) × 100

Body weight gain (BWG,%) = [(final weight–initial weight)/initial weight] × 100

Feed conversion ratio (FCR) = dry weight of feed consumed (g)/live weight gain (g).

Specific growth rate (SGR,% day^–1^) = [(Ln final weight–Ln initial weight)/duration] × 100

The statistical analyses were performed using the software SPSS 20.0. All data were presented by means ± standard error of the mean (SEM, *n* = 3). One-way ANOVA was used to detect the differences among the treatments. When significant differences (*p* < 0.05) were identified among treatments, Duncan’s multiple range test was used to compare the means of the treatments.

### Ethics Statement

The present study was carried out in strict accordance with the recommendations in the ethical guidelines of EU Directive 2010/63/EU for animal experiments.

## Results

### Growth Performance, Survival, and Feed Utilization

Growth performance, including final body weight (FBW), body weight gain (BWG), as well as the specific growth rate (SGR), was significantly affected by dietary Ast supplementation levels (Table 3). FBW, BWG, and SGR of shrimp fed with Ast including diets (B, C, D, E, and F), were significantly (*p* < 0.05) higher than the one fed the control diet, but without significant difference (*p* > 0.05) with shrimp fed with high Ast levels (E and F). The increasing dietary Ast levels improved shrimp survival and nutrient utilization of diet (FCR), and there were significant (*p* < 0.05) differences in D, E, and F treatments compared with the control.

**TABLE 3:** Growth parameters and nutrient utilization of *P. monodon* fed with experimental diets for 56 days.^#^

**Parameters**	**Treatments**					
	**A**	**B**	**C**	**D**	**E**	**F**
Survival (%)	80.00 ± 0.81^a^	81.00 ± 0.57^ab^	82.5 ± 0.95^abc^	83.00 ± 0.57^bc^	83.00 ± 1.29^bc^	84.5 ± 0.50^c^
FBW (g)	13.55 ± 0.16^a^	14.54 ± 0.09^b^	15.30 ± 0.06^c^	15.59 ± 0.15^c^	16.16 ± 0.13^d^	16.46 ± 0.05^d^
BWG (%)	351.58 ± 5.51^a^	384.67 ± 3.28^b^	409.92 ± 2.17^c^	419.58 ± 5.03^c^	438.75 ± 4.41^d^	448.67 ± 1.90^d^
SGR (%/day)	2.51 ± 0.02^a^	2.63 ± 0.01^b^	2.72 ± 0.01^c^	2.75 ± 0.01^c^	2.81 ± 0.01^d^	2.84 ± 0.01^d^
FCR	1.79 ± 0.02^c^	1.76 ± 0.02^c^	1.64 ± 0.01^b^	1.61 ± 0.02^b^	1.53 ± 0.02^a^	1.49 ± 0.01^a^

*^#^FBW, final body weight; BWG, body weight gain; SGR, specific growth rate; FCR, feed conversion ratio. Data are expressed as mean ± SEM from triplicate groups.*

*Mean values in the same row with different letters are significantly different (*p* < 0.05).*

### Shrimp Whole-Body Composition

The effect of different dietary levels of Ast on proximate composition of shrimp whole body (% dry basis) is shown in Table 4. The crude protein, crude lipid, ash, and moisture contents in the whole body did not show any statistical differences among all the treatments (*p* > 0.05).

**TABLE 4:** Proximate composition of shrimp whole body.

**Parameters** ^a^	**Treatments**					
	**A**	**B**	**C**	**D**	**E**	**F**
**Basal composition (%)**						
Crude protein	61.12 ± 0.45	61.51 ± 0.80	62.60 ± 0.41	60.55 ± 0.82	60.34 ± 0.37	60.07 ± 0.14
Crude lipid	4.67 ± 0.07	4.39 ± 0.05	4.95 ± 0.17	4.41 ± 0.03	4.11 ± 0.10	4.60 ± 0.18
Ash	12.54 ± 0.45	13.37 ± 0.23	12.77 ± 0.10	12.36 ± 0.44	12.87 ± 0.33	13.04 ± 0.29
Moisture	78.67 ± 0.32	78.12 ± 0.75	78.30 ± 0.41	79.03 ± 0.36	79.06 ± 0.52	79.28 ± 0.65

*^*a*^Data are expressed as mean ± SEM from triplicate groups.*

### Total Carotenoid Contents and Carotenoid Composition Analysis in Different Tissues

**FIGURE 1:**
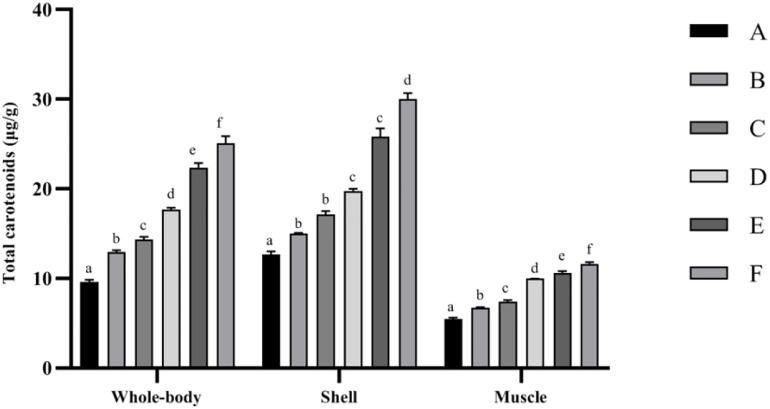
Total carotenoid contents of *Penaeus monodon* fed with different astaxanthin levels for 56 days. Data are expressed as mean ± SEM from triplicate groups. Different letters indicate significant (*p* < 0.05) differences among various bars. S.E.M., standard error of mean.

Total carotenoid contents in whole-body, shell, and muscle samples of the studied shrimp are presented in Figure 1. The highest total carotenoid content of the whole body, shell, and muscle tissues was determined in the F treatment, while the lowest was observed in the control (*p* < 0.05).

The contents of six main kinds of carotenoids, including Ast, zeaxanthin, canthaxanthin, β-cryptoxanthin, echinenone, and β-carotene detected in the shrimp whole body, shell, and muscle are listed in Table 5. The shrimp fed the Ast-supplemented diets (B–F) showed markedly (*p* < 0.05) increasing trends in Ast concentrations with dietary Ast supplementation in the whole body, shell, and muscle tissues. The contents of canthaxanthin and β-cryptoxanthin showed continuous increasing trends with the increase in dietary Ast levels in different tissues. Zeaxanthin was only detected in the muscle, and the whole body belongs to the treatment with high-level Ast supplementation.

**TABLE 5:** Carotenoid contents (μg/g) analysis in tissues of *Penaeus monodon.*^#^

**Parameters**	**Treatments**					
	**A**	**B**	**C**	**D**	**E**	**F**
**Whole body**						
Astaxanthin	4.2 ± 0.15^a^	5.13 ± 0.24^ab^	6.24 ± 0.08^b^	8.88 ± 0.12^c^	11.35 ± 0.40^d^	12.01 ± 0.84^d^
Zeaxanthin	ND	ND	ND	0.13 ± 0.02^a^	0.33 ± 0.01^b^	0.26 ± 0.06^b^
Canthaxanthin	0.44 ± 0.03^a^	0.68 ± 0.08^ab^	0.80 ± 0.04^b^	0.88 ± 0.09^b^	1.03 ± 0.02^bc^	1.42 ± 0.17^c^
β-cryptoxanthin	0.38 ± 0.03^a^	0.50 ± 0.11^ab^	0.60 ± 0.23^ab^	0.64 ± 0.07^ab^	0.76 ± 0.03^b^	1.30 ± 0.02^c^
Echinenone	0.68 ± 0.08^a^	1.18 ± 0.20^ab^	0.89 ± 0.01^a^	1.26 ± 0.02^ab^	2.06 ± 0.16^b^	2.76 ± 0.35^c^
β-carotene	1.06 ± 0.40^a^	1.46 ± 0.23^ab^	1.92 ± 0.07^bc^	2.58 ± 0.19^cd^	3.05 ± 0.13^d^	5.32 ± 0.01^e^
**Shell**						
Astaxanthin	5.13 ± 0.10^a^	6.78 ± 0.13^b^	7.65 ± 0.03^c^	8.81 ± 0.04^d^	11.21 ± 0.20^e^	12.01 ± 0.38^e^
Zeaxanthin	ND	ND	ND	ND	ND	ND
Canthaxanthin	0.66 ± 0.03^a^	0.66 ± 0.02^a^	0.87 ± 0.03^ab^	1.02 ± 0.03^b^	1.50 ± 0.02^c^	1.54 ± 0.03^c^
β-cryptoxanthin	0.57 ± 0.15^a^	1.20 ± 0.16^b^	1.80 ± 0.05^b^	2.28 ± 0.04^c^	2.52 ± 0.09^c^	2.85 ± 0.08^c^
Echinenone	1.38 ± 0.27^a^	1.47 ± 0.07^a^	1.95 ± 0.08^ab^	2.88 ± 0.06^b^	3.39 ± 0.03^c^	4.27 ± 0.28^d^
β-carotene	2.79 ± 0.40^ab^	2.19 ± 0.11^a^	3.66 ± 0.07^b^	4.25 ± 0.21^b^	5.34 ± 0.41^c^	6.73 ± 0.10^d^
**Muscle**						
Astaxanthin	1.34 ± 0.04^a^	1.75 ± 0.04^a^	2.51 ± 0.01^b^	3.18 ± 0.04^c^	3.38 ± 0.07^c^	3.70 ± 0.45^c^
Zeaxanthin	ND	ND	ND	ND	0.08 ± 0.01^a^	0.27 ± 0.03^b^
Canthaxanthin	0.18 ± 0.01^a^	0.21 ± 0.04^a^	0.33 ± 0.01^ab^	0.39 ± 0.04^ab^	0.42 ± 0.03^ab^	0.54 ± 0.39^b^
β-cryptoxanthin	0.56 ± 0.04^a^	0.65 ± 0.16^a^	0.70 ± 0.13^ab^	0.79 ± 0.04^ab^	1.03 ± 0.06^b^	1.11 ± 0.08^b^
Echinenone	0.85 ± 0.08^a^	1.17 ± 0.10^ab^	1.44 ± 0.03^ab^	2.03 ± 0.01^ab^	2.31 ± 0.25^b^	2.57 ± 0.12^b^
β-carotene	0.85 ± 0.23^a^	1.44 ± 0.10^b^	1.49 ± 0.14^b^	2.26 ± 0.17^c^	2.70 ± 0.06^c^	2.78 ± 0.20^c^

*^#^Data are expressed as mean ± SEM from triplicate groups. Mean values in the same row with different letters are significantly different (*p* < 0.05).*

*ND, not detected. Shell: including shrimp carapace, telson, and uropod.*

### Antioxidant Parameters

The results obtained from the determination of antioxidant activities in shrimp hepatopancreas are shown in Table 6. The T-AOC significantly increased in Ast-supplemented treatments compared with the control, while SOD, CAT, and MDA decreased significantly (*p* < 0.05).

**TABLE 6:** Hepatopancreatic antioxidant status of *P. monodon* fed with different astaxanthin levels.^#^

**Parameters**	**Treatments**					
	**A**	**B**	**C**	**D**	**E**	**F**
SOD (U/mg protein)	39.62 ± 0.97^b^	37.83 ± 0.88^b^	32.29 ± 0.89^a^	32.05 ± 1.34^a^	31.57 ± 0.55^a^	30.74 ± 0.59^a^
T-AOC (U/mg protein)	1.19 ± 0.01^a^	1.31 ± 0.06^b^	1.32 ± 0.01^b^	1.39 ± 0.04^bc^	1.45 ± 0.05^c^	1.48 ± 0.02^c^
MDA (nmol/mg protein)	30.01 ± 0.19^d^	24.90 ± 0.76^c^	24.00 ± 0.45^bc^	22.07 ± 0.87^b^	18.38 ± 0.64^a^	17.29 ± 0.64^a^
CAT (U/mg protein)	9.20 ± 0.39^d^	8.85 ± 0.03^d^	7.26 ± 0.08^c^	5.57 ± 0.12^b^	5.09 ± 0.18^b^	4.03 ± 0.07^a^

*^#^Data are expressed as mean ± SEM from triplicate groups. Mean values in the same row with different letters are significantly different (*p* < 0.05).*

*SOD, superoxide dismutase; T-AOC, total antioxidant capacity; MDA, malondialdehyde; CAT, catalase.*

### Hemolymph-Immunological Parameters

The hemolymph-immunological results of *P. monodon* are presented in Table 7. AKP increased significantly (*p* < 0.05) in dietary Ast treatments, while no more significance (*p* > 0.05) was detected among D–F treatments. The AST showed a continuous decreasing trend with the increase in dietary Ast levels. Ast-supplemented levels over 61.5 mg/kg diet (D treatment) showed significantly higher values compared with the control. By contrast, dietary Ast significantly (*p* < 0.05) decreased ALT activities in the hemolymph of shrimp.

**TABLE 7:** Hemolymph-immunological parameters of *P. monodon* fed with experimental diets.^#^

**Parameters**	**Treatments**					
	**A**	**B**	**C**	**D**	**E**	**F**
AST (U/mg protein)	18.74 ± 0.89^d^	17.92 ± 0.49^cd^	17.52 ± 0.63^bcd^	15.31 ± 0.60^abc^	15.08 ± 1.29^ab^	14.42 ± 0.85^a^
ALT (U/mg protein)	26.12 ± 1.17^d^	15.25 ± 0.31^c^	14.89 ± 0.47^c^	12.18 ± 1.05^b^	10.40 ± 0.49^ab^	8.79 ± 0.73^a^
AKP (U/mg protein)	5.66 ± 0.07^a^	7.38 ± 0.14^b^	8.56 ± 0.22^bc^	9.67 ± 0.12^cd^	10.62 ± 0.73^d^	10.93 ± 0.63^d^

*^#^Data are expressed as mean ± SEM from triplicate groups. Mean values in the same row with different letters are significantly different (*p* < 0.05). AST, aspartate aminotransferase; ALT, alanine aminotransferase; AKP, alkaline phosphatase.*

### Stress Resistance Test

**FIGURE 2:**
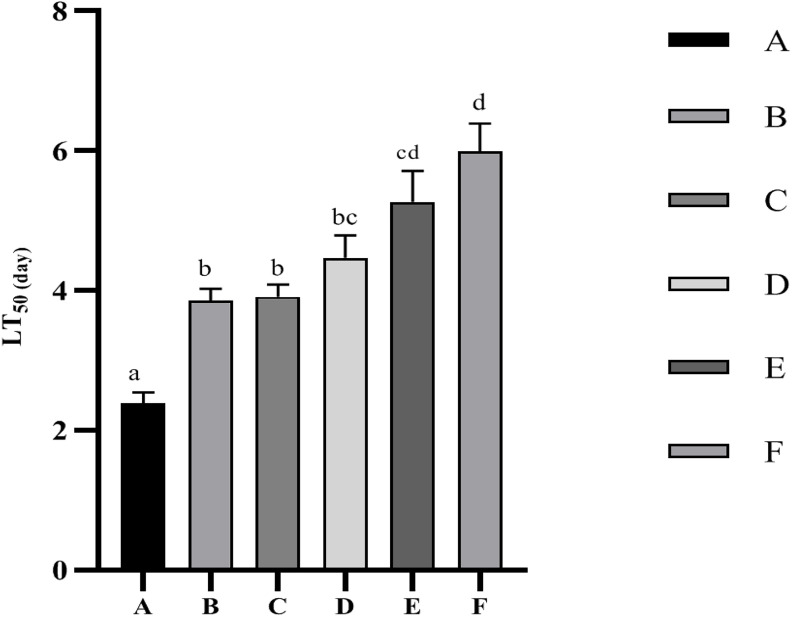
Time to 50% mortality (LT_50_) of *P. monodon* after infected with *Vibrio parahaemolyticus*. Data are expressed as mean ± SEM from triplicate groups. Different letters indicate significant (*p* < 0.05) differences among various bars.

The LT_50_ values for the *Vibrio parahaemolyticus* infecting stress resistance test are shown in Figure 2. The LT_50_ values were significantly (*p* < 0.05) increased by dietary supplementation of Ast. The best performance was given by F treatment, while no significant (*p* > 0.05) difference was observed compared with treatment E.

### Intestinal Microbiota Comparison

**FIGURE 3:**
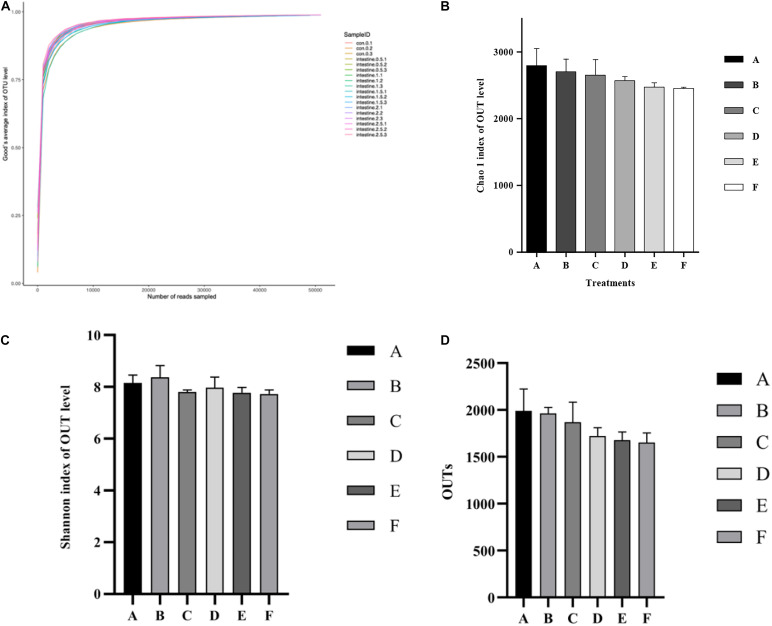
Analysis of alpha diversity comparisons with the microbial communities in intestine of shrimp fed with different astaxanthin levels for 56 days. **(A)** Good’s coverage curve; **(B)** Chao species richness index; **(C)** Shannon’s diversity index; **(D)** OTUs richness comparison index for A, B, C, D, E, and F intestinal samples. Data are expressed as mean ± SEM from triplicate groups. Different letters indicate significant (*p* < 0.05) differences among various bars. OTUs, operational taxonomic units.

The alpha diversity statistics and OTUs of the intestinal microbiota in *P. monodon* are shown in Figure 3. The mean of Good’s coverage (Figure 3A) for each treatment approached 99%. The Shannon and Chao 1 results could reflect the bacterial diversity and richness information of the intestine samples (Figures 3B,C). These two parameters did not show any significant (*p* > 0.05) differences among all the treatments. Although OTUs (Figure 3D) decreased with the increase in dietary Ast levels, no significant (*p* > 0.05) difference was detected among all the treatments.

**FIGURE 4:**
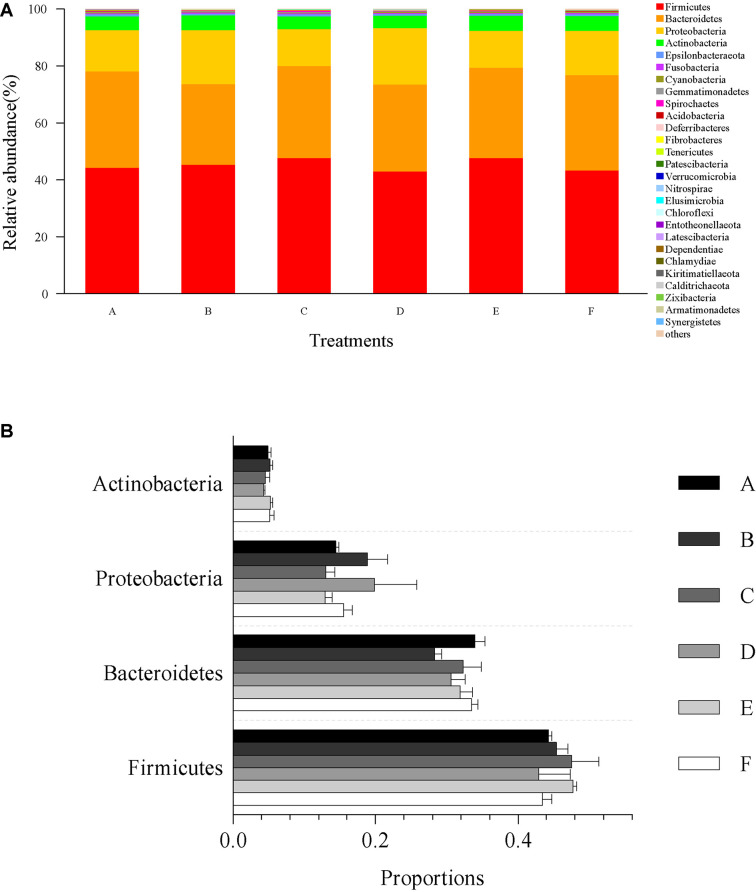
Relative abundances of dominant phyla and comparison with bacterial abundances in the intestine at the phylum level. **(A)** Relative abundances of dominant phyla (top 30) from all classifiable sequencing data. Unclassified phyla with relative abundances lower than 1% were assigned as “others.” **(B)** The abundance comparison with four dominant microbial community members at the phylum level, including Firmicutes, Bacteroidetes, Proteobacteria, and Actinobacteria. Data are expressed as mean ± SEM from triplicate groups.

The microbial community composition and abundance of all intestinal samples at the phylum level are presented in Figure 4. The most dominant microbial community members at the phylum level, Firmicutes, Bacteroidetes, Proteobacteria, and Actinobacteria accounted for 45.23, 31.66, 15.82, and 4.87% of the total microbial community, respectively (Figure 4A). No significant differences were observed in relative abundance of Firmicutes (*p* = 0.67), Bacteroidetes (*p* = 0.27), Proteobacteria (*p* = 0.41), and Actinobacteria (*p* = 0.61) among all treatments (Figure 4B).

**FIGURE 5:**
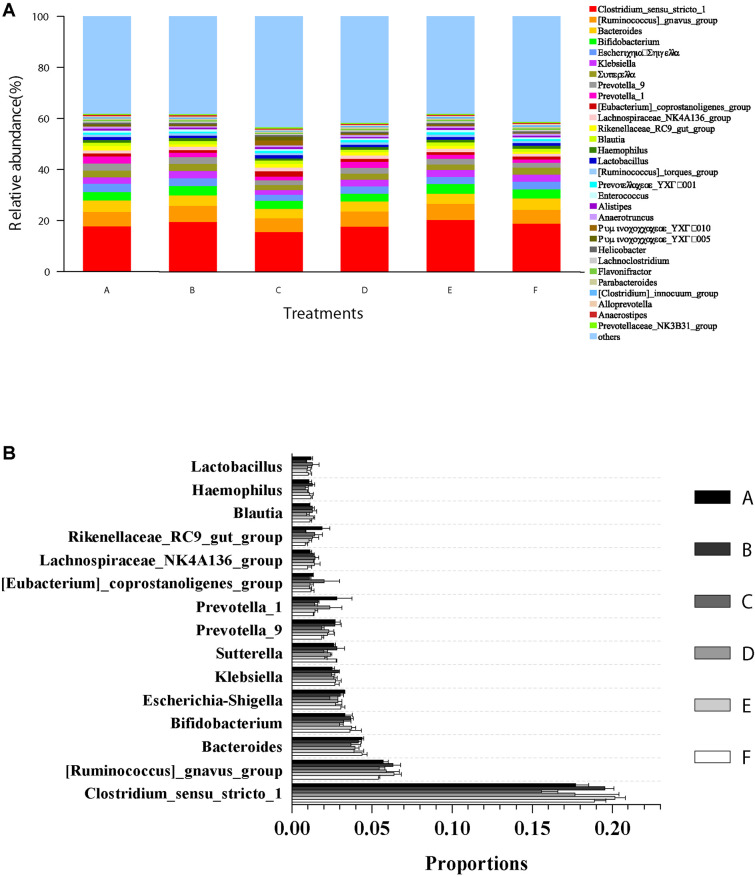
Relative abundances of dominant genera and comparison with bacterial abundances in the intestine at the genus level. **(A)** Relative abundances of dominant genera (top 30) from all classifiable sequencing data. Unclassified genera with relative abundances lower than 1% were assigned as “others.” **(B)** The abundance comparison with 15 most abundant genera in shrimp intestine among all the treatments. Data are expressed as mean ± SEM from triplicate groups.

The microbial community composition and abundance of all intestinal samples at the genus level are presented in Figure 5. The top 15 genera in classifiable sequences were *Clostridium*, *Ruminococcus*, *Bacteroides*, *Bifidobacterium*, *Escherichia–Shigella*, *Klebsiella*, *Sutterella*, *Prevotella*, *Prevotella*, *Eubacterium*, *Lachnospiraceae*, *Rikenellaceae*, *Blautia*, *Haemophilus*, and *Lactobacillus* (Figure 5A). The ANOVA test analysis results did not show any significant differences (*p* > 0.05) in the relative abundance of these genera (Figure 5B) among all the treatments.

## Discussion

### Biological Performance

Carotenoids are primarily used as pigments sources in aquaculture for improving product quality (Shahidi and Brown, 1998). Besides this crucial role, carotenoids also play an essential role in enhancing nutrient utilization and ultimately improving the growth and survival of aquatic animals (Niu et al., 2012; Zhang et al., 2013). In the aquaculture industry, the cost of feed represents about 50–60% of a total operational cost depending on production scale and cultivation methods (Lim et al., 2017). Therefore, the beneficial role of Ast as a critical nutritive additive for tremendous growth and survival is necessary to minimize the production cost fluctuation caused by its high price (Niu et al., 2009; Wang et al., 2018a). Currently, commercial production of Ast is dominated by synthetic Ast because of its relatively lower production costs compared with the other alternatives (Saini and Keum, 2018). Over the years, an increasing number of reports have revealed the positive correlations between dietary synthetic Ast supplementation and growth performance and survival, or either of them in different species of aquatic animals (Wade et al., 2017; Wang et al., 2019). A few studies have been conducted to evaluate the *P. rhodozyma* Ast on growth performance due to its production capacity limitation. Recently, the increasing demand of consumers for biotechnological production makes synthetic pigment less desirable, and Ast obtained from biological sources perform a long-term gain potential in the global market. Furthermore, the Ast form (3R, 3′R optical isomer) in *P. rhodozyma* is supposed to own greater bioavailability (Bjerkeng et al., 2007). Previous studies have demonstrated the beneficial effects of dietary synthetic and algal Ast in a variety of shrimp species with evidence that Ast supplementation could boost weight gain and improve survival in *P. monodon* (Niu et al., 2012), *Litopenaeus vannamei* (Flores et al., 2007; Niu et al., 2009), *Macrobrachium rosenbergii* (Kumar et al., 2009), *Paralithodes camtschaticus* (Daly et al., 2013), and *Marsupenaeus japonicus* (Wang et al., 2018b). The current study supported the positive effect of yeasty Ast supplementation on the growth and survival performance in *P. monodon*. Combined, these data consistently suggest that dietary Ast as a nutritional factor is important in stimulating aquatic animals’ growth and survival. However, some controversies were found among studies on *L. vannamei* (Ju et al., 2011), *M. japonicus* (Yamada et al., 1990; Chien and Jeng, 1992), and *Eriocheir sinensis* (Long et al., 2017; Jiang et al., 2020), which demonstrated that growth and survival of juveniles were similar regardless of whether including Ast in the diet or not. These inconsistent conclusions may be explained by different carotenoid requirements among aquatic animal species, culture condition (indoor or not, exogenous carotenoids intake or not, culture period, temperature, and density), the composition of feed (basal carotenoid content in the diet), and state of animal health.

Likewise, dietary Ast supplementation between 25 and 100 mg/kg diet was found to remarkably boost weight gain of *P. monodon* but without affecting survival rate (Wade et al., 2015). In another study, *L. vannamei* performed considerable growth increasing on 125 and 150 mg/kg levels of Ast intake than those fed with comparatively lower levels of Ast (25, 50, 75, and 100 mg/kg diet), but survival was not affected (Zhang et al., 2013). Significant correlations have been observed between tissue carotenoid concentration and survival (Chien and Jeng, 1992). Wade et al. (2017) suggested that survival would not be affected when body carotenoid contents are above a certain level, otherwise, they would be compromised below that level without carotenoid supplementation. Thus, when initial tissue carotenoid levels are high enough during the feeding trial, further carotenoids supplementation allows improved growth rather than survival improvement (Niu et al., 2014). Growth parameters, including final body weight (FBW), body weight gain (BWG), and specific growth rate (SGR), mainly reflect the retention or deposition of nutrients from feed into the body but do not precisely equate to the health status of the aquatic animal (Du and Turchini, 2021). Moreover, the positive effect on survivability by dietary Ast appears to be closely linked to its proposed antioxidant capacity, immune response regulation, and stress alleviative property (Niu et al., 2012; Wade et al., 2017). Thus, non-specific immune response and disease resistance against *V. parahaemolyticus* parameters were complemented in the current study to achieve a more reliable assessment of healthy growth of juveniles fed with *P. rhodozyma* Ast.

### Whole-Body Composition and Total Carotenoid Contents of Shrimp

The whole-body composition results are consistent with the findings of Niu et al. (2009) and Zhang et al. (2013) where the carotenoid supplementation seemed to not affect the proximate composition of shrimp whole-body.

Aquatic animals, including crustaceans, cannot synthesize carotenoids *de novo*, must obtain carotenoids through their diets (Matsuno, 2001). Numerous studies have demonstrated that dietary carotenoids could increase the deposition of carotenoids in the tissues (Ju et al., 2011; Wang et al., 2018b). In the current study, total carotenoid content in various tissues (Figure 1) significantly increased with the increasing Ast supplemented levels, which showed similar trends on growth, antioxidant capacity, and immune response performance. This phenomenon implied that crustaceans generally accumulate carotenoids in tissues, including hepatopancreas, shell, muscle, and ovaries, that would be metabolized when dietary supply is low to optimize health status during developmental processes or in response to environmental circumstances (Wade et al., 2017; Babin et al., 2019).

As the best-established function, there is no doubt that shrimp dietary with Ast supplementation would achieve desired red color due to increased Ast concentration in shell (Shahidi and Brown, 1998). Jiang et al. (2020) reported that exoskeleton Ast contents are correlated with the redness (*a*^∗^) value of crustaceans on cooking, which reflects the Ast concentrations in tissues. The current study detected increased Ast concentration in shell with the increasing dietary Ast levels as expected. Moreover, dietary Ast significantly affected the zeaxanthin, canthaxanthin, and β-carotene levels in tissues. Yamada et al. (1990) stated that decapod crustaceans have carotenoid metabolic conversion capacity in their internal organs. The accumulation of specific carotenoids also implies their corresponding particular function in different tissues. Moreover, an increased presence of Ast in shrimp muscle (Table 5) could confer many benefits for human health due to its potent antioxidant activity, such as anti-inflammatory, anti-aging, immune system boosting, anti-cancer, and sun proofing (Lim et al., 2017).

### Antioxidant Capacity and Non-specific Immune Response

High-density shrimp aquaculture operations are frequently subject to various physical and surrounding environment stressors involving grading, transport, crowding, hypoxia, temperature fluctuation, and high ammonia nitrogen loading, ultimately leading to bodily physiological dysfunction and immune suppression that are susceptible toward pathogenic invasions (Liu et al., 2016). Invertebrates lack an adaptive immune system, mainly relying on innate immune responses as the first line to defend against invaders (Yan et al., 2020). AKP is a crucial phosphatase that regulates phosphorylation and dephosphorylation processes involving animal growth, nutrient transport, and pathogen storage in the lysosome (Zhu et al., 2021). In the current study, the AKP activities in hemolymph significantly increased with the increase in dietary Ast levels, indicating a positive effect of dietary Ast on the immunity of *P. monodon.* This finding agreed with previous studies on *Eriocheir sinensis* (Jiang et al., 2020). Changes in the ALT and AST enzymes levels in hemolymph always indicate the physiological state of hepatopancreas cells (Ettefaghdoost and Haghighi, 2021). Generally, kept at a low level, they are significantly increased in the hemolymph when the hepatopancreas are severely damaged. There is a similarity in the mentioned studies (Niu et al., 2012) with the current one on the significant reduction of ALT and AST activities in Ast-supplemented treatments (Table 7). Furthermore, a study on *Oncorhynchus mykiss* indicated decreased serum ALT and AST fed with *P. rhodozyma* (Nakano et al., 1999). Combined results indicated the significant role of Ast on the status improvements of hepatopancreatic cells in *P. monodon.*

As a vital and multifunctional organ, the hepatopancreas of crustaceans performed critical functions, including metabolism, detoxification, and secretion (Vogt, 2019). The hepatopancreatic antioxidant enzymes were used as biomarkers to display the effect of dietary Ast on the antioxidant status of *P. monodon.* The immune and antioxidant systems in crustaceans are two crucial physiological mechanisms that modulate immunological function, ensuring optimal cellular functions and confer physiological improvements (Zhu et al., 2021). SOD is a major antioxidant enzyme that acts as a scavenger for superoxide radicals that result from oxidative stress (Chien et al., 2003). CAT is widely distributed in several tissues to consume the peroxides as an intracellular H_2_O_2_ scavenger (Hugo, 1984). In general, the activities of SOD and CAT would be upregulated to quench reactive oxygen species (ROS) produced during stress (Duan et al., 2015). Studies on *Macrobrachium nipponense* (Ettefaghdoost and Haghighi, 2021), *P. monodon* (Niu et al., 2014), and *E. sinensis* (Jiang et al., 2020) showed a significant reduction of these two enzymes in treatments containing Ast compared with control, which were consistent with the present study. T-AOC represents a comprehensive indicator of the antioxidant status of an individual (Zhang et al., 2013). MDA is the primary product of lipid peroxidation and can destroy the structure and function of cells. Assaying the activity of this enzyme indicates lipid peroxidation (Esterbauer and Cheeseman, 1990). In the current study, dietary Ast significantly increased T-AOC and reduced the MDA in hepatopancreas, which supports the result on SOD and CAT, indicating a lower oxidative stress level in shrimp dietary Ast supplementation. The positive performance on antioxidant parameters of Ast could attribute to its extreme capacity to scavenge oxygen free radicals and prevention of peroxidation of PUFAs in tissues (Wade et al., 2017).

### Susceptibility of Shrimp to *Vibrio parahaemolyticus*

Various disease outbreaks caused by vibriosis due to environmental deterioration have occurred within booming *P. monodon* aquaculture, leading to significant economic impacts worldwide (Duan et al., 2015). Several studies have reported that *V. parahaemolyticus* could induce acute hepatopancreatic necrosis (AHPND) by causing a compromised immunity and hepatopancreatic damage of shrimp (Zhu et al., 2021). Dietary yeasty Ast significantly increased resistance to *V. parahaemolyticus* according to the LT_50_ results in the current study, which may be contributing to the anti-bacterial effects, which indicated that dietary Ast could suppress *V. parahaemolyticus* infection. However, *V. parahaemolyticus* is a conditional pathogen: the pathogenicity is triggered by physical stressors that disrupt the delicate balance between aquatic animals and surrounding environments (Pang et al., 2019). Thus, improved immune status by inducing AST, ALT, AKP, and SOD in the shrimp by dietary Ast is also one of the primary reasons to defend against pathogenic infections in the current study.

### Intestinal Microbiota Analysis

Bacterial microbiota analysis in the intestines of farmed aquatic animals has been paid attention to reveal its relationships and shrimp health (Zhu et al., 2021). Intestinal bacteria and bacterial metabolites directly affect the nutrient absorption and immunity of the host (Ayiku et al., 2020). The current study is the first time to discuss the effect of dietary Ast supplementation on the microbial community in the intestine of *P. monodon*. No significant differences were observed in the intestinal bacterial richness and diversities among all the treatments. Consistent with the research on *L. vannamei* (Ayiku et al., 2020; Wang et al., 2021), Firmicutes, Bacteroidetes, Proteobacteria, and Actinobacteria performed the predominant phyla in the intestine of shrimp. These bacteria have been well known to play a significant role in the physiological metabolism of the host (Sun et al., 2020). While the relative abundances of these four significant phyla could be influenced by the rearing environment and the basal nutrient composition of the diet, compositions of the intestinal bacteria at the phylum levels were quite similar among different studies. The predominant genera in the current study differ from the results obtained in other organisms: for example, Wang et al. (2021) revealed that *Candidatus Bacilloplasma, Vibrio, Lactobacillus*, *Spongiimonas*, and *Flavirhabdus* were the predominant genera in the intestine of *L. vannamei.* In the intestines of *P. monodon*, Chaiyapechara et al. (2012) found the dominant bacterial genera were *Vibrio*, *Photobacterium*, *Aeromonas*, or *Propionigenium*, which conclude that the intestinal bacteria in genus levels varied significantly and were distinct from their rearing environment. No significant differences were detected on the dominant phyla and genera among all treatments in the current. Combined results indicated that dietary Ast supplementation did not significantly affect bacterial diversity, dominant bacterial communities, or bacterial community similarities in the intestine of *P. monodon*.

## Conclusion

Dietary Ast from *P. rhodozyma* could provide desired growth performance, antioxidant status, non-specific immune response, resistance to disease, and carotenoid contents of *P. monodon.* Moreover, the promising performance of *P. rhodozyma* Ast as the natural source could provide an alternative to synthetic Ast in the *Penaeus monodon* culture industry.

## Data Availability Statement

The datasets presented in this study can be found in online repositories. The names of the repository/repositories and accession number(s) can be found below: BioProject accession number: PRJNA761235.

## Author Contributions

WW drafted the manuscript. ML finished the feeding trial and chemical analysis. SF helped to analyze the data. YX made the figures. MW put forward relevant experimental guidance. GY and XH designed the research. QL worked on manuscript revision. All authors read and gave final approval of the manuscript.

## Conflict of Interest

GY was employed by the company Beijing Dabeinong Technology Group Co., Ltd. The remaining authors declare that the research was conducted in the absence of any commercial or financial relationships that could be construed as a potential conflict of interest.

## Publisher’s Note

All claims expressed in this article are solely those of the authors and do not necessarily represent those of their affiliated organizations, or those of the publisher, the editors and the reviewers. Any product that may be evaluated in this article, or claim that may be made by its manufacturer, is not guaranteed or endorsed by the publisher.

## Footnotes

1
www.oebiotech.com
